# 1x8 Gy versus 5x4 Gy for metastatic epidural spinal cord compression: a matched-pair study of three prognostic patient subgroups

**DOI:** 10.1186/s13014-018-0968-3

**Published:** 2018-02-08

**Authors:** Dirk Rades, Antonio J. Conde-Moreno, Jon Cacicedo, Theo Veninga, Barbara Segedin, Karmen Stanic, Volker Rudat, Steven E. Schild

**Affiliations:** 1 Department of Radiation Oncology, University of Lübeck, Ratzeburger Allee 160, 23538 Lübeck, Germany; 2 Department of Radiation Oncology, Consorcio Hospital Provincial de Castellón, Castellón, Spain; 3 Department of Radiation Oncology, Cruces University Hospital, Vizcaya, Barakaldo, Spain; 4 Department of Radiotherapy, Dr. Bernard Verbeeten Institute, Tilburg, Netherlands; 5 Department of Radiotherapy, Institute of Oncology Ljubljana, Ljubljana, Slovenia; 6 Department of Radiation Oncology, Saad Specialist Hospital, Al-Khobar, Saudi Arabia; 7 Department of Radiation Oncology, Mayo Clinic Scottsdale, Scottsdale, AZ USA

## Abstract

**Background:**

This study provides separate comparisons of 1 × 8 Gy to 5 × 4 Gy for metastatic epidural spinal cord compression (MESCC) in patients with poor, intermediate and favorable survival prognoses.

**Methods:**

Patients receiving 1 × 8 Gy were matched to patients receiving 5 × 4 Gy for age, gender, performance status, tumor type, involved vertebrae, other bone metastases, visceral metastases, interval between tumor diagnosis and MESCC, ambulatory status and time developing motor deficits. From a study including patients with poor (*N* = 156) or intermediate (*N* = 86) survival prognoses, subgroup analyses were performed. Furthermore, 232 new patients with favorable prognoses matched the same way were included.

**Results:**

In poor prognoses patients, 6-month survival rates were 10% after 1 × 8 Gy and 6% after 5 × 4 Gy (*p* = 0.38); in-field reRT rates in few patients alive at 6 months were 15 and 2% (*p* = 0.16). In intermediate prognoses patients, 6-month survival rates were 49% after 1 × 8 Gy and 58% after 5 × 4 Gy (*p* = 0.30). ReRT rates at 6 months were 23 and 13% (*p* = 0.25). In favorable prognoses patients, 6-month survival rates were 89% after 1 × 8 Gy and 91% after 5 × 4 Gy. ReRT rates at 6 months were 14 and 3% (*p* = 0.007). In no subgroup, RT regimen had a significant impact on motor function.

**Conclusions:**

Since in patients with poor prognoses, outcomes after 1 × 8 Gy and 5 × 4 Gy were not significantly different, 1 × 8 Gy may be an option. In patients with intermediate prognoses, a trend was found in favor of 5 × 4 Gy. In patients with favorable prognoses, need for in-field reRT was greater after 1 × 8 Gy.

## Background

Metastatic epidural spinal cord compression (MESCC) is an oncologic emergency that is most often treated with radiotherapy (RT) alone [1, 2]. Many patients irradiated for MESCC have poor or intermediate survival prognoses. Generally, radiation oncologists attempt to minimize the number of palliative radiation fractions, ideally to a single fraction mostly of 8 Gy or 10 Gy. One prerequisite for administration of single-fraction regimens would be that these regimens provide comparable outcomes to multi-fraction regimens in terms of improvement of motor deficits, need for in-field reRT for MESCC and survival (OS). During recent years, one matched-pair study and two randomized trials were reported that compared single-fraction RT with 1 × 8 Gy or 1 × 10 Gy to short-course multi-fraction RT with 5 × 4 Gy over 1 week [3–5]. According to the previous matched-pair study 1 × 8 Gy was not inferior to 5 × 4 Gy for effect of RT on motor function, need for reRT for MESCC in the irradiated part of the spine, and survival [3]. This matched-pair study included patients with poor and intermediate survival prognoses based on a validated survival score [6]. In a randomized trial presented in abstract form in 2014, 1 × 10 Gy was not inferior to 5 × 4 Gy with respect to response to RT defined as improvement or no further progression of motor deficits and ambulatory status at 5 weeks following RT [4]. According to the results of a phase III trial, which was presented in abstract form in 2017 and compared 1 × 8 Gy to 5 × 4 Gy, 1 × 8 Gy was not inferior to 5 × 4 Gy with respect to walking ability assessed 8 weeks following randomization (not following RT) [5]. However, both randomized trials were limited to poor prognoses patients [4, 5]. The matched-pair study was limited to patients with poor or intermediate survival prognoses and did not differentiate between these two cohorts [3]. Thus, it is not clear whether the non-inferiority of single-fraction RT is limited to patients with a poor survival prognosis or should also be a viable option for intermediate or favorable prognoses patients. Therefore, the present study has been performed which is the first study that looked separately at the relative merits of single-fraction RT for MESCC in patients with poor, intermediate and favorable survival prognoses.

## Methods

According to our previous matched-pair study 1 × 8 Gy was not inferior to 5 × 4 Gy for effect of RT on motor function, need for re-treatment for MESCC in the irradiated part of the spine, and survival. The decision regarding the need of re-treatment was based on the presence of clinical symptoms (new or progressive motor deficits) and corresponding findings on magnetic resonance imaging showing recurrent or progressive MESCC in the parts of the spine that were previously irradiated. This endpoint is very similar to local control of MESCC. Data regarding remineralization of osteolytic bone and the rate of pathological fractures of vertebral bodies were not available and, therefore, not included in this study.

That study included patients with poor and intermediate survival prognoses according to a validated survival score [6]. For this scoring system, three major prognostic groups with 20–30, 31–35 and 36–45 points, respectively, were defined with 6-month OS rates of 9, 48 and 93%, respectively. The present study was approved by the local ethic committee (University of Lübeck) and performed in accordance with the precepts established by the Helsinki Declaration. Criteria for inclusion, diagnostic procedures and details of radiotherapy were previously described [3]. Patients receiving 1 × 8 Gy were matched (1:1) to patients receiving 5 × 4 Gy (over 1 week) for 10 characteristics including age, gender, performance status, primary tumor type, number of involved vertebrae, other bone metastases, visceral metastases, interval between tumor diagnosis and MESCC, pre-RT ambulatory status, and time developing motor deficits prior to RT (Tables 1 and 2).

**Table 1:** Distribution of characteristics in patients with poor survival prognoses

	8 Gy × 1 N patients (%)	4 Gy × 5 N patients (%)
Age		
≤65 years (*N* = 78)	39 (50)	39 (50)
≥66 years (*N* = 78)	39 (50)	39 (50)
Gender		
Female (*N* = 36)	18 (23)	18 (23)
Male (*N* = 120)	60 (77)	60 (77)
ECOG Performance status		
1–2 (*N* = 26)	13 (17)	13 (17)
3–4 (*N* = 130)	65 (83)	65 (83)
Type of primary tumor		
Breast cancer (*N* = 12)	6 (8)	6 (8)
Prostate cancer (*N* = 36)	18 (23)	18 (23)
Myeloma/lymphoma (*N* = 0)	0 (0)	0 (0)
Lung cancer (*N* = 46)	23 (29)	23 (29)
Unknown primary (*N* = 32)	16 (21)	16 (21)
Other tumors (*N* = 30)	15 (19)	15 (19)
Involved vertebrae (n)		
1–2 (*N* = 34)	17 (22)	17 (22)
≥3 (*N* = 122)	61 (78)	61 (78)
Other bone metastases		
No (*N* = 34)	17 (22)	17 (22)
Yes (*N* = 122)	61 (78)	61 (78)
Visceral metastases		
No (*N* = 36)	18 (23)	18 (23)
Yes (*N* = 120)	60 (77)	60 (77)
Interval from tumor diagnosis to MESCC		
≤ 15 months (*N* = 136)	68 (87)	68 (87)
> 15 months (*N* = 20)	10 (13)	10 (13)
Pre-RT ambulatory status		
Not ambulatory (*N* = 110)	55 (71)	55 (71)
Ambulatory (*N* = 46)	23 (29)	23 (29)
Time developing motor deficits		
1–7 days (*N* = 110)	55 (71)	55 (71)
8–14 days (*N* = 30)	15 (19)	15 (19)
> 14 days (*N* = 16)	8 (10)	8 (10)

**Table 2:** Distribution of characteristics in patients with intermediate survival prognoses

	8 Gy × 1 N patients (%)	4 Gy × 5 N patients (%)
Age		
≤65 years (*N* = 30)	15 (35)	15 (35)
≥66 years (*N* = 56)	28 (65)	28 (65)
Gender		
Female (*N* = 26)	13 (30)	13 (30)
Male (*N* = 60)	30 (70)	30 (70)
ECOG Performance status		
1–2 (*N* = 32)	16 (37)	16 (37)
3–4 (*N* = 54)	27 (63)	27 (63)
Type of primary tumor		
Breast cancer (*N* = 12)	6 (14)	6 (14)
Prostate cancer (*N* = 34)	17 (40)	17 (40)
Myeloma/lymphoma (*N* = 4)	2 (5)	2 (5)
Lung cancer (*N* = 12)	6 (14)	6 (14)
Unknown primary (*N* = 4)	2 (5)	2 (5)
Other tumors (*N* = 20)	10 (23)	10 (23)
Involved vertebrae (n)		
1–2 (*N* = 34)	17 (40)	17 (40)
≥3 (*N* = 52)	26 (60)	26 (60)
Other bone metastases		
No (*N* = 34)	17 (40)	17 (40)
Yes (*N* = 52)	26 (60)	26 (60)
Visceral metastases		
No (*N* = 50)	25 (58)	25 (58)
Yes (*N* = 36)	18 (42)	18 (42)
Interval from tumor diagnosis to MESCC		
≤ 15 months (*N* = 44)	22 (51)	22 (51)
> 15 months (*N* = 42)	21 (49)	21 (49)
Pre-RT ambulatory status		
Not ambulatory (*N* = 44)	22 (51)	22 (51)
Ambulatory (*N* = 42)	21 (49)	21 (49)
Time developing motor deficits		
1–7 days (*N* = 24)	12 (28)	12 (28)
8–14 days (*N* = 24)	12 (28)	12 (28)
> 14 days (*N* = 38)	19 (44)	19 (44)

Patients were assessed for motor function prior to RT, directly after RT, at 1 month following RT, and additionally if they developed progressive or new symptoms of MESCC. In the patients who had progressive or new motor deficits after RT, magnetic resonance imaging was performed. Scans were reviewed by experienced neuro-radiologists to differentiate between non-pathological and pathological (i.e. due progression or a recurrence of MESCC) fractures. To evaluate the effect of RT on motor deficits, motor function was assessed prior to RT and at 1 month following RT using a 5-point scale [7]: 0 = normal strength; 1 = ambulatory without aid, 2 = ambulatory with aid, 3 = not ambulatory, 4 = paraplegia. Improvement or deterioration of motor function was defined as a change of one or more points.

In the present study, additional subgroup analyses were performed for patients with poor survival prognoses (*n* = 156) and patients with an intermediate prognoses (*n* = 86). Furthermore, 232 new patients with favorable prognoses [6] were matched 1:1 as describes above and separately analyzed the same way. Patient characteristics of the three cohorts are shown in Table 1 (poor prognoses), Table 2 (intermediate prognoses) and Table 3 (favorable prognoses).

**Table 3:** Distribution of characteristics in patients with favorable survival prognoses

	8 Gy × 1 N patients (%)	4 Gy × 5 N patients (%)
Age		
≤65 years (*N* = 120)	60 (52)	60 (52)
≥66 years (*N* = 112)	56 (48)	56 (48)
Gender		
Female (*N* = 118)	59 (51)	59 (51)
Male (*N* = 114)	57 (49)	57 (49)
ECOG Performance status		
1–2 (*N* = 192)	96 (83)	96 (83)
3–4 (*N* = 40)	20 (17)	20 (17)
Type of primary tumor		
Breast cancer (*N* = 90)	45 (39)	45 (39)
Prostate cancer (*N* = 86)	43 (37)	43 (37)
Myeloma/lymphoma (*N* = 28)	14 (12)	14 (12)
Lung cancer (*N* = 12)	6 (5)	6 (5)
Unknown primary (*N* = 2)	1 (1)	1 (1)
Other tumors (*N* = 14)	7 (6)	7 (6)
Involved vertebrae (n)		
1–2 (*N* = 104)	52 (45)	52 (45)
≥3 (*N* = 128)	64 (55)	64 (55)
Other bone metastases		
No (*N* = 124)	62 (53)	62 (53)
Yes (*N* = 108)	54 (47)	54 (47)
Visceral metastases		
No (*N* = 212)	106 (91)	106 (91)
Yes (*N* = 20)	10 (9)	10 (9)
Interval from tumor diagnosis to MESCC		
≤ 15 months (*N* = 58)	29 (25)	29 (25)
> 15 months (*N* = 174)	87 (75)	87 (75)
Pre-RT ambulatory status		
Not ambulatory (*N* = 28)	14 (12)	14 (12)
Ambulatory (*N* = 204)	102 (88)	102 (88)
Time developing motor deficits		
1–7 days (*N* = 12)	6 (5)	6 (5)
8–14 days (*N* = 40)	20 (17)	20 (17)
> 14 days (*N* = 180)	90 (78)	90 (78)

### Statistical methods

Time to in-field reRT and death were referenced from the last day of RT and calculated with the Kaplan-Meier-method. Differences between Kaplan-Meier curves were calculated with the log-rank test. A difference between the curves was considered significant if the *p*-value was < 0.05. The univariate analyses of motor function were performed with the ordered-logit-model (− 1 = deterioration, 0 = no further progression, 1 = improvement of motor deficits), because the data were ordinal. Again, the results were considered significant if the p-value was < 0.05.

## Results

### Patients with poor survival prognoses

**Fig. 1:**
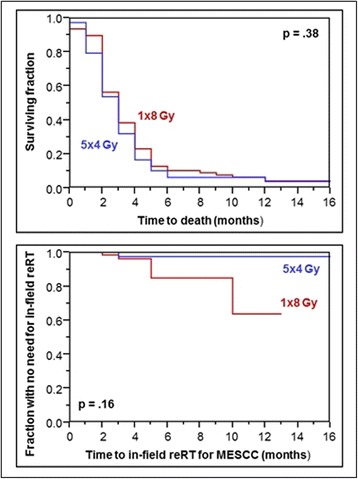
Comparison of 1 × 8 Gy and 5 × 4 Gy with respect to survival (top) and the need for in-field reRT for MESCC (bottom) in patients with poor survival prognoses

In this subgroup (*N* = 156), median survival was 3 months, and OS rates at 6 and 12 months were 8 and 4%, respectively. RT regimen had no significant impact on OS (*p* = 0.38, Fig. 1). Six-month OS rates were 10% after 1 × 8 Gy and 6% after 5 × 4 Gy, respectively; 12-month OS rates were 4 and 4%, respectively. In-field reRT for MESCC at 6 and 12 months was required in 15 and 36% of patients, respectively, after 1 × 8 Gy, and in 2 and 2% of patients, respectively, after 5 × 4 Gy (*p* = 0.16, Fig. 1). When interpreting the results regarding the need for in-field reRT, one has to be aware that only 18 and 8 patients, respectively, were alive at 6 and 12 months following RT. Five patients receiving 1 × 8 Gy and 2 patients receiving 5 × 4 Gy died within 1 month following RT. Thus, 73 and 76 patients, respectively, were evaluable for the RT-effect on motor function. The RT regimen had no significant impact on motor function (*p* = 0.23). Improvement was found in 7 patients (10%) after 1 × 8 Gy and 6 patients (8%) after 5 × 4 Gy, respectively. No further progression was observed in 41 (56%) and 53 (70%) patients, respectively, deterioration in 25 (34%) and 17 (22%) patients, respectively.

#### Patients with intermediate survival prognoses

**Fig. 2:**
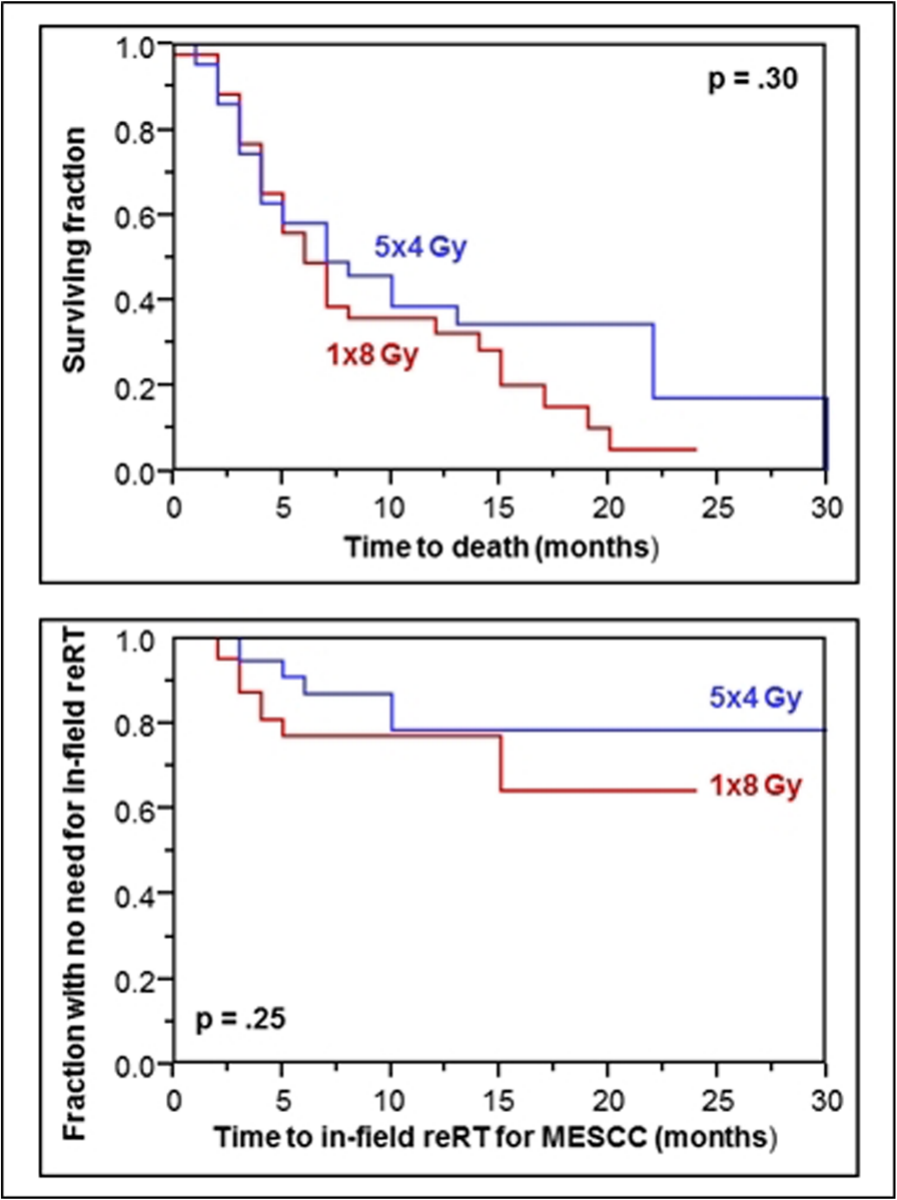
Comparison of 1 × 8 Gy and 5 × 4 Gy with respect to survival (top) and the need for in-field reRT for MESCC (bottom) in patients with intermediate survival prognoses

In this subgroup (*N* = 86), median survival time was 7 months. OS rates at 6 and 12 months were 53 and 35%, respectively. Again, the RT regimen had no significant impact on OS (*p* = 0.30, Fig. 2). Six-month OS rates were 49% after 1 × 8 Gy and 58% after 5 × 4 Gy, respectively; 12-month OS rates were 32 and 39%, respectively. Rates of in-field reRT for MESCC at 6 and 12 months were 23 and 23%, respectively, after 1 × 8 Gy, and 13 and 22%, respectively, after 5 × 4 Gy (*p* = 0.25, Fig. 2). One patient receiving 1 × 8 Gy died within 1 month following RT, and 42 patients were evaluable for the RT-effect on motor function in the 1 × 8 Gy-group. The RT regimen had no significant impact on motor function (*p* = 0.40). Improvement was found in 12 patients (29%) after 1 × 8 Gy and 17 patients (39.5%) after 5 × 4 Gy, respectively. No further progression was observed in 24 (57%) and 20 (46.5%) patients, respectively, deterioration in 6 (14%) and 6 (14%) patients, respectively.

#### Patients with favorable survival prognoses

**Fig. 3:**
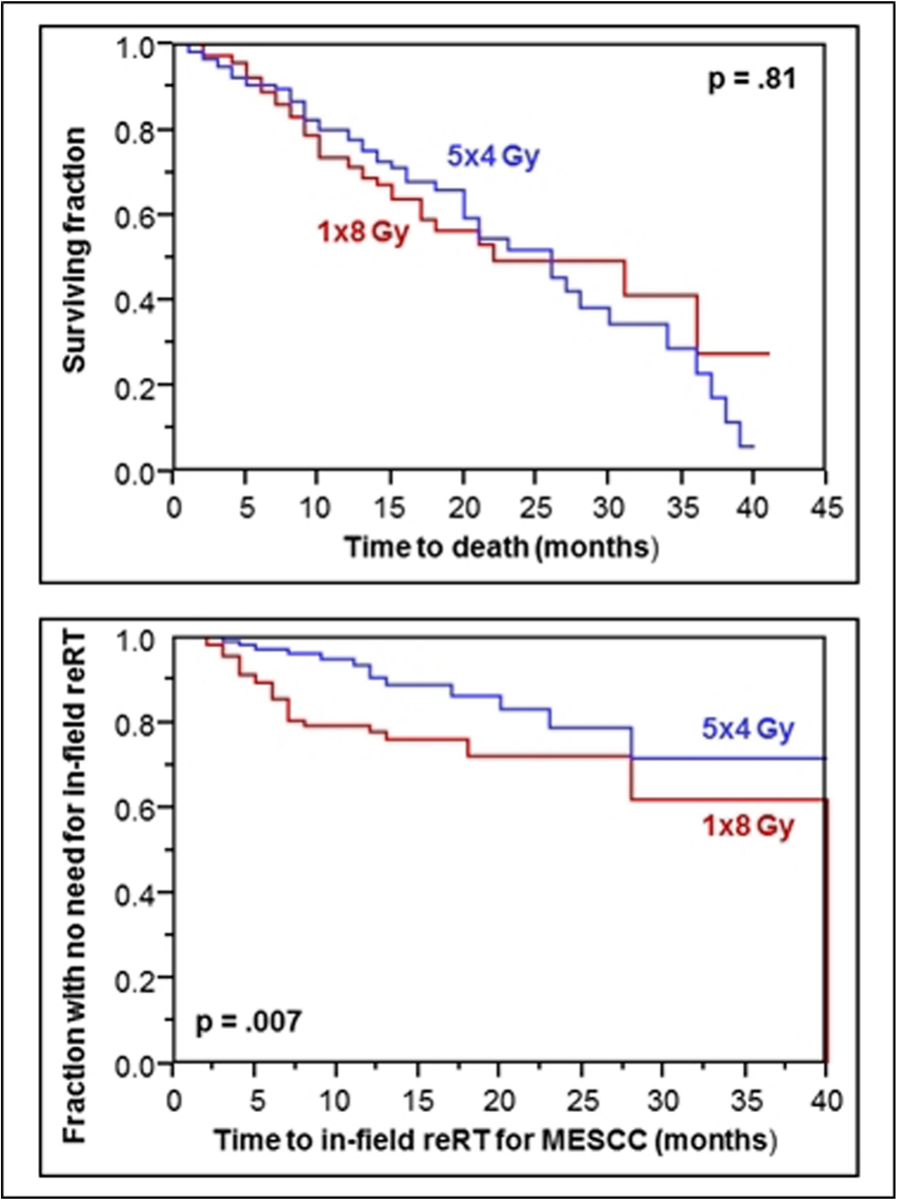
Comparison of 1 × 8 Gy and 5 × 4 Gy with respect to survival (top) and the need for in-field reRT for MESCC (bottom) in patients with favorable survival prognoses

In this additional group (*N* = 232), median survival time was 26 months, and OS rates at 6 and 12 months were 90 and 74%, respectively. The RT regimen was not significantly associated with OS (*p* = 0.81, Fig. 3). Six-month OS rates were 89% after 1 × 8 Gy and 91% after 5 × 4 Gy, respectively, and 12-month OS rates were 71 and 78%, respectively. Rates of in-field reRT for MESCC at 6 and 12 months were 14 and 22%, respectively, after 1 × 8 Gy, and 3 and 9%, respectively, after 5 × 4 Gy (*p* = 0.007, Fig. 3). All patients of this group were evaluable for the RT-effect on motor function. The RT regimen had no significant impact on motor function (*p* = 0.22). Improvement was found in 44 patients (38%) after 1 × 8 Gy and 54 patients (47%) after 5 × 4 Gy, respectively. No further progression was observed in 67 (58%) and 57 (49%) patients, respectively, deterioration in 5 (4%) and 5 (4%) patients, respectively.

## Discussion

Most patients with MESCC receive RT, either alone or following decompressive surgery. However, surgery is generally indicated for selected patients with a good performance status, a relatively favorable survival prognosis, and MESCC from metastases of a solid tumor and involvement of no more than one spinal segment. These patients only account for 10–15% of all patients with MESCC [1, 2, 8]. Thus, the majority of patients with MESCC are treated with RT alone. For MESCC, a considerable number of dose-fractionation programs are used worldwide ranging from single-fraction programs to multi-fraction programs with up to 20 fractions over 4 weeks [1, 2]. Since treatment is palliative and may be associated with discomfort for the often debilitated patients, the number of fractions of RT should be kept low as long as it does not jeopardize outcomes. Ideally, MESCC patients would be treated with only one fraction resulting in fewer visits to the radiation oncology department and possibly less time in the hospital. Three studies were presented since 2014 that compared single-fraction with 1 × 8 Gy or 1 × 10 Gy to multi-fraction RT with 5 × 4 Gy over 1 week [3–5]. In 2014, a randomized non-inferiority trial compared 1 × 10 Gy to 5 × 4 Gy over 1 week in 115 eligible patients with MESCC and a poor survival prognosis [4]. The rates of improvement or at least stability of mobility (overall response) at 5 weeks following RT were 79% after 1 × 10 Gy and 68% after 5 × 4 Gy, respectively (*p* > 0.05). The mobility deterioration-free survival times were 1.4 months and 1.4 months, respectively. Thus, 1 × 10 Gy was similar in efficacy to 5 × 4 Gy for patients with MESCC and poor survival prognoses. The other randomized trial, which was presented in 2017, compared 1 × 8 Gy to 5 × 4 Gy in 688 patients irradiated for MESCC [5]. The median survival in the entire cohort of this trial was poor with only about 3 months. Primary endpoint was ambulatory status (ambulatory with or without aid vs. not ambulatory) at 8 weeks following randomization, not following RT. The non-inferiority margin was given as 11%. Due to the poor survival, only 340 patients (49%) were evaluable at 8 weeks. The ambulatory rates were 70% (114 patients) after 1 × 8 Gy and 73% (129 patients) after 5 × 4 Gy, respectively (difference not significant). Both randomized trials were performed in patients with MESCC and poor survival prognoses. Therefore, it remains unclear whether 1 × 8 Gy was similar in efficacy to 5 × 4 Gy for patients with intermediate or favorable survival prognoses. The matched-pair study published in 2015 compared 1 × 8 Gy and 5 × 4 Gy in 242 patients with poor or intermediate survival prognoses. Endpoints included OS, need for in-field reRT for MESCC and effect of RT on motor function [3]. OS rates at 6 months and 12 months following RT were 24 and 9%, respectively after 1 × 8 Gy compared to 35 and 13% after 5 × 4 Gy, respectively (*p* = 0.65). ReRT for an in-field recurrence of MESCC at 6 months and 12 months was required 18 and 30%, respectively after 1 × 8 Gy and in 9 and 22%, respectively after 5 × 4 Gy (*p* = 0.11). The effect of RT on motor function was also not significantly different with 1 × 8 Gy and 5 × 4 Gy (*p* = 0.21) with improvement rates of 17 and 23%, respectively. Thus, 1 × 8 Gy appeared statistically similar to 5 × 4 Gy with respect to all three investigated endpoints.

This previous matched-pair study did not differentiate between patients with a poor survival prognosis and those patients with an intermediate prognosis [3]. Thus, it was uncertain whether 1 × 8 Gy was similar in efficacy to 5 × 4 Gy in both cohorts individually. Therefore, the present matched-pair study was performed that includes separate subgroup analyses of the previous study for patients with poor prognoses and those with intermediate prognoses, respectively. According to the results of these subgroup analyses, 1 × 8 Gy appeared statistically similar to 5 × 4 Gy with respect to OS, need for in-field reRT and effect on motor function in patients with poor prognoses. Thus, 1 × 8 Gy appears a reasonable option for patients with poor prognoses. The intermediate prognoses group was much smaller (*N* = 86), and may, therefore, be limited in terms of statistical power. The results of this study showed a trend in favor of 5 × 4 Gy in this subgroup, although statistical significance was not reached. Therefore, one should be quite reserved using 1 × 8 Gy in this subgroup and should use the single-fraction regimen only in carefully selected patients.

The question whether 1 × 8 Gy would be a viable option for patients with favorable survival prognoses could not be answered with the subgroup analyses of our previous study [3]. Therefore, additional analyses were performed in a new cohort of patients with favorable prognoses. These 232 patients were matched 1:1 for the same 10 characteristics as the two subgroups from the previous matched-pair study to ensure quality and comparability [3]. It was determined that 1 × 8 Gy was also similar to 5 × 4 Gy with respect to OS and effect on motor function. However, the need for an in-field reRT for a subsequent episode of MESCC was significantly greater after 1 × 8 Gy than after 5 × 4 Gy. Because an in-field recurrence of MESCC is often difficult to treat, 1 × 8 Gy appears not appropriate for favorable survival prognoses patients. This finding agrees with the results of previous studies in non-matched cohorts that suggested a dose-effect relation for freedom from an in-field recurrence of MESCC following irradiation [9, 10]. However, although the present study included strict matching criteria (1:1-matching for 10 characteristics), it is based on retrospective data and, therefore, bears a risk of including hidden selection biases. However, additional randomized trials will take several years and cannot be expected in the near future. Furthermore, a randomized trial investigating 1 × 8 Gy for MESCC in patients with favorable survival prognoses will likely not be possible, since the present study and previous studies in unmatched cohorts suggested 1 × 8 Gy to be significantly inferior to multi-fraction regimens with respect to freedom from an in-field recurrence of MESCC requiring reRT [9, 10]. Furthermore, it has been previously demonstrated that fractionated RT results in more pronounced remineralization of the osteolytic bone and likely in prevention of pathological fractures than single-fraction RT, which may be critical in weight-bearing regions of the spine [11]. Therefore, 1 × 8 Gy should not be used in patients with osteolytic metastases, who are at risk of experiencing a pathological fracture. Moreover, since only 30 patients (6%) in the present study had a primary tumor considered less radiosensitive, the results cannot be generalized to these patients, who may require RT with higher biologically effective doses beyond 40 Gy or upfront decompressive surgery [12–16]. Another group of patients who could benefit from higher doses are patients with oligometastatic disease, which can for example be defined as involvement of 1–3 vertebrae by MESCC and absence of other bone and visceral metastases [16, 17].

## Conclusion

In patients with poor survival prognoses, 1 × 8 Gy was not significantly inferior to 5 × 4 Gy with respect to need for in-field reRT for MESCC, OS and effect of RT on motor function. Therefore, 1 × 8 Gy may be a reasonable option for this group. In patients with intermediate prognoses, a trend was found in favor of 5 × 4 Gy. Therefore, 1 × 8 Gy should be limited to carefully selected patients of this group. In patients with favorable prognoses, the need for in-field reRT was significantly greater after 1 × 8 Gy than after 5 × 4 Gy. Therefore, 1 × 8 Gy should not be used for patients with favorable prognoses.

## Abbreviations

MESCC: Metastatic epidural spinal cord compression
OS: Overall survival
reRT: Re-irradiation
RT: Radiotherapy
